# The Ependymal Region Prevents Glioblastoma From Penetrating Into the Ventricle via a Nonmechanical Force

**DOI:** 10.3389/fnana.2021.679405

**Published:** 2021-06-07

**Authors:** Kaishu Li, Haimin Song, Chaohu Wang, Zhiying Lin, Guozhong Yi, Runwei Yang, Bowen Ni, Ziyu Wang, Taichen Zhu, Wanghao Zhang, Xiran Wang, Zhifeng Liu, Guanglong Huang, Yawei Liu

**Affiliations:** ^1^Department of Neurosurgery, Nanfang Hospital, Southern Medical University, Guangzhou, China; ^2^Department of Neurosurgery, The Sixth Affiliated Hospital of Guangzhou Medical University, Qingyuan, China; ^3^The Laboratory for Precision Neurosurgery, Nanfang Hospital, Southern Medical University, Guangzhou, China

**Keywords:** glioblastoma, subventricular zone, ventricle, penetration, ependymal

## Abstract

**Background:**

Intraventricular penetration is rare in glioblastoma (GBM). Whether the ependymal region including the ependyma and subventricular zone (SVZ) can prevent GBM invasion remains unclear.

**Methods:**

Magnetic resonance imaging (MRI) and haematoxylin–eosin (HE) staining were performed to evaluate the size and anatomical locations of GBM. Binary logistic regression analysis was used to assess the correlation between tumor-ependyma contact, ventricle penetration and clinical characteristics. Cell migration and invasion were assessed via Transwell assays and an orthotopic transplantation model.

**Results:**

Among 357 patients with GBM, the majority (66%) showed ependymal region contact, and 34 patients (10%) showed ventricle penetration of GBM. GBM cells were spread along the ependyma in the orthotopic transplantation model. The longest tumor diameter was an independent risk factor for GBM-ependymal region contact, as demonstrated by univariate (OR = 1.706, *p* < 0.0001) and multivariate logistic regression analyses (OR = 1.767, *p* < 0.0001), but was not associated with ventricle penetration. Cerebrospinal fluid (CSF) could significantly induce tumor cell migration (*p* < 0.0001), and GBM could grow in CSF. Compared with those from the cortex, cells from the ependymal region attenuated the invasion of C6 whether cocultured with C6 or mixed with Matrigel (*p* = 0.0054 and *p* = 0.0488). Immunofluorescence analysis shows a thin gap with GFAP expression delimiting the tumor and ependymal region.

**Conclusion:**

The ependymal region might restrict GBM cells from entering the ventricle via a non-mechanical force. Further studies in this area may reveal mechanisms that occur in GBM patients and may enable the design of new therapeutic strategies.

## Introduction

Glioblastoma (GBM) is the most common and aggressive primary malignant brain tumor in adults (Grossman et al., 2009). The high degree of infiltration is one of the hallmarks of GBM (Li et al., 2019; Vollmann-Zwerenz et al., 2020). Invasion and dissemination of tumor cells into surrounding brain tissues results in mortality in GBM patients. Nevertheless, dissemination of GBM in the ventricles occurs very rarely.

The ependyma lines the ventricles of the vertebrate brain and forms a protective barrier (Jimenez et al., 2014). It is natural to conjecture that GBM cell infiltration is blocked by the ependyma due to mechanical effects. However, the ependyma is only a monolayer of multiciliated epithelial cells (Garcia-Verdugo et al., 2002), which does not support this statement.

The subventricular zone (SVZ) is located at the border of the lateral ventricles (Sanai et al., 2005). As the largest neurogenic niche in the adult mammalian brain, the SVZ has attracted extensive attention from scholars (Doetsch et al., 1999; Sanai et al., 2004; Cayre et al., 2009; Codega et al., 2014; Lee et al., 2018; Li et al., 2020). Many studies suggested that the SVZ plays an important role in the progression of GBM (Sanai et al., 2004; Liu et al., 2016; Qin et al., 2017; Mistry et al., 2017a; Lee et al., 2018; Altmann et al., 2019; Mistry et al., 2019). Analyses of the survival of patients with GBM revealed that it is crucial for the tumor to have a direct connection to the SVZ (Goffart et al., 2017; Mistry et al., 2017a; Mistry, 2019). However, little attention has been given to why GBMs rarely penetrate the SVZ or ependyma into the ventricles.

Thus, this study will provide evidence regarding the prohibition of GBM cell invasion by the ependymal region and explore whether this region provides mechanical protection.

## Materials and Methods

### Patient Cohort and the MRI Characteristics

We performed an Institutional Review Board–approved retrospective review of 357 patients with GBM who underwent surgery, radiotherapy, and chemotherapy at our institution from January 2010 through April 2020. Patients were included in the study if their preoperative and postoperative MRI results were available on the picture archiving and communication system of our hospital for review. Patients were classified as involving the ependymal region if the contrast-enhancing lesion contacted the lining of the ventricle (≤ 2 mm) (Sanai et al., 2005; Mistry et al., 2017a,b, 2019). This study was based on retrospective data and represents a single-center experience: all information was assessed from the data available in the medical records. A single-center sample may not be representative of the general patient population.

### Cell Culture

U87, C6 and GL261 cells were obtained from the American Type Culture Collection (ATCC). All cell lines were routinely cultured in high-glucose DMEM (Gibco, Thermo Fisher, United States) growth medium supplemented with 10% foetal bovine serum (FBS) (Gibco, Thermo Fisher, United States) at 37°C in a humidified 5% CO_2_ incubator (Heal Force HF90, China) buffer (Yi et al., 2019).

### Animals

Specific-pathogen-free Sprague-Dawley rats weighing 350 g and C57BL/6 mice weighing 20 g were used for this study. Animals were maintained in accordance with the Association for Assessment and Accreditation of Laboratory Animal Care criteria, and all studies were approved by the institutional animal care committee.

### Dissection of the Rat Ependymal Region and Cortex

Isolation and of the ependymal region and cortex and preparation of the single-cell suspensions were performed according to the protocols reported by Azari et al. (2010).

### Transwell Experiments

Cell invasion was determined using a Transwell Matrigel invasion assay in 24-well Transwell units (Costar, Coring Incorporated, United States). Each chamber was filled with 20 μg Matrigel (Thermo Fisher Scientific, Inc.) and 1 × 10^4^ cells. The lower chambers were filled with 500 μL culture media. After termination of treatment, the non-invading cells were removed with cotton swabs. The inserts were removed from the top chambers, washed with PBS, fixed and stained with Giemsa dye. The invaded cells were counted in five random fields under a light microscope. The procedure used for cell migration was similar to that used for invasion, except that Matrigel was not added to the upper chamber. The experiment was repeated three times with three replicates.

### Intracranial Tumor Cell Injection

The mice were fixed in a stereotactic apparatus (Model 900; Kopf Instruments, Tujunga, CA) for injection of tumor cells into the right hemisphere. The injection point was 3 mm behind the coronal suture and 2 mm lateral to the sagittal suture. The injection point of the ventricle was 3 mm behind the coronal suture and 1 mm lateral to the sagittal suture. A 10 μL microliter syringe with a flat tip was used to inject 2 × 10^5^ cells suspended in 6 μL PBS. Each group included 8 mice. Only the mice that survived for 2 weeks after the operation and were confirmed to have successful establishment of the intracranial xenograft tumor model by MRI were included in the analysis. MRI was performed using a small animal scanner (PharmaScan70/16 US).

### Histological Examination

The specimens were fixed in 10% formalin and embedded in paraffin, and 4 μm-thick sections were obtained. HE staining and immunohistochemical examination were performed.

### Immunofluorescence of Tissue Sections

The GFAP rabbit polyclonal antibody (CST, Catalog Number: 80788), EMA mouse monoclonal polyclonal antibody (ZSGB-bio, Catalog Number: ZM-0095), β3-tubulin mouse monoclonal antibody (CST, Catalog Number: 5568), goat polyclonal secondary antibody to mouse IgG-H&L (Alexa Fluor® 488, Abcam, Catalog Number: ab150113), donkey polyclonal secondary antibody to rabbit IgG-H&L (Alexa Fluor® 647, Abcam, Catalog Number: ab150075) and mounting medium with DAPI (Abcam, ab104139) were used. Fluorescence microscopy was performed on an OLYMPUS BX63 fluorescence microscope.

### Statistical Analysis

Statistical analyses were performed using GraphPad Prism 8. For comparison of two groups, two-tailed unpaired Student’s *t*-tests were performed with a confidence level of 95%. For comparisons across multiple groups, two-tailed unpaired Student’s *t*-test with Holm–Sidak correction or one-way ANOVA with Bonferroni correction for multiple comparisons was used where noted. The statistical significance threshold was set at *P* ≤ 0.05. The risk ratio (RR)/odds ratio (OR) was used for outcome estimation whenever appropriate with a 95% confidence interval (CI) in logistic regression.

## Results

### Patient Cohort

The clinical characteristics of the 357 patients are shown in Table 1. Of the 357 patients, 40 (11%) were over 65, 230 (64%) were male, 208 (58%) had wild-type IDH, 196 (55%) had a longest diameter over 5 cm, 237 (66%) had tumor-ependymal region contact (ependymal +), 120 (34%) had tumors separated from the ependymal region (ependymal-), 53 (15%) had MGMT expression over one plus, 226 (63%) had EGFR expression over one plus, 158 (44%) had VEGF expression over one plus, and 141 (39%) had Ki67 expression over 40% (Table 1).

**TABLE 1:** Characteristics of the study population.

Characteristic	All (*n* = 357)	Ependymal + (*n* = 237, 66%)	Ependymal - (*n* = 120, 34%)
**Age**			
Average (years, mean ± *SD*)	47.2 (± 15.1)	46.9 (± 15.3)	47.7 (± 14.7)
< 65 years	317 (89%)	210 (59%)	107 (30%)
≥65 years	40 (11%)	27 (8%)	13 (4%)
**Gender**			
Female	127 (36%)	84 (24%)	43 (12%)
Male	230 (64%)	153 (43%)	77 (22%)
**IDH status**			
IDH-mutant	60 (17%)	40 (11%)	20 (6%)
IDH-wild type	208 (58%)	141 (39%)	67 (19%)
IDH-unknown	89 (25%)	56 (16%)	33 (9%)
**Longest diameter**			
Average (cm, mean ± *SD*)	5.2 (± 1.7)	5.6 (± 1.6)	4.3 (± 1.5)
<5 cm	161 (45%)	77 (22%)	84 (24%)
≥5 cm	196 (55%)	160 (45%)	36 (10%)
**Intraventricular metastasis**			
Not entered	304 (85%)	184 (52%)	120 (34%)
Entered	34 (10%)	34 (10%)	0 (0%)
Unidentified	19 (5%)	19 (5%)	0 (0%)
MGMT expression			
≤+	304 (85%)	202 (57%)	102 (29%)
>+	53 (15%)	35 (10%)	18 (5%)
**EGFR expression**			
≤+	131 (37%)	89 (25%)	42 (12%)
>+	226 (63%)	148 (41%)	78 (22%)
**VEGF expression**			
≤+	199 (56%)	137 (38%)	62 (17%)
>+	158 (44%)	100 (28%)	58 (16%)
**Ki67**			
<40%	216 (61%)	143 (40%)	73 (20%)
≥40%	141 (39%)	94 (26%)	47 (13%)

### MRI and Pathological Analysis

**FIGURE 1:**
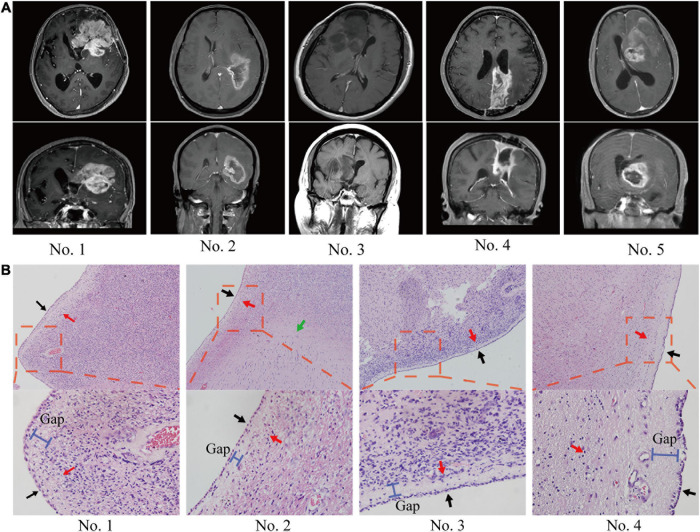
The anatomical relationship between the tumor and SVZ/ependymal region was determined by MRI and HE staining. **(A)** On MRI, glioblastoma spread along the ependymal and pushed the lateral ventricle, but there was no sign of breaking through the lateral ventricle. **(B)** Pathological biopsy also indicated that glioblastoma cells spread along the ependymal but did not enter the lateral ventricle wall. Red arrows, tumor; black arrows, ependymal; green arrows, normal tissue; blue bar, subependymal gap.

The anatomical relationship between the tumor and ependymal region was determined by two methods: a statement of lateral ventricle entry documented in the operative note and/or preoperative MRI. In most cases, GBM spread along the ependymal region was verified not only radiographically but also by direct visualization (Figure 1A). HE staining confirmed that tumor cells grew along the ventricle wall and that the ependyma were separated from the tumor by a thin gap (Figure 1B). MRI images of the patients with ependymal entry are shown in Supplementary Figure 1A, and MRI images of the patients with ventricle entry are displayed in Supplementary Figure 1B.

### Analysis of Clinical Characteristics

**FIGURE 2:**
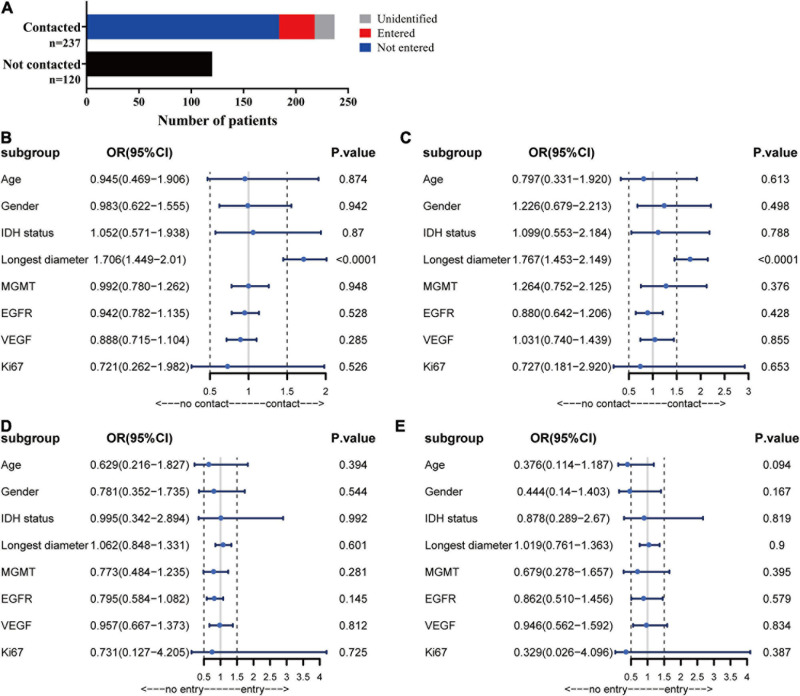
Analysis of MRI results. **(A)** Statistics for the relationship between tumor location and ependymal region. **(B)** Forest plot showing that a high longest tumor diameter was a risk factor for ependymal region contact, whereas there was no correlation with patient age, sex, IDH status, MGMT, EGFR, VEGF or Ki67. **(C)** The results showed that the ventricular entry risk was not correlated with age, sex, longest tumor diameter, IDH status, MGMT, EGFR, VEGF or Ki67. **(D)** All the characteristics have no correlation with the lateral ventricle penetration of GBM in the univariate binary logistics analyses. **(E)** All the characteristics have no correlation with the lateral ventricle penetration of GBM in the multivariate binary logistics analyses.

In the patients with ependymal +, 184 (52%) of the patients had no MRI evidence of a tumor entering the ventricle but showed signs of tumor compression. Ventricle entry occurred in only 34 (10%) patients (Table 1 and Figure 2A). The patients with ventricle entry had no pathological biopsy verification. Univariate binary logistic regression analyses suggested that no significant correlation was observed between ependymal + status and patient age, sex or IDH status, but a significant association was observed between ependymal + status and longest tumor diameter (OR = 1.706; 95% CI, 1.449–2.01; *p* < 0.001) (Figure 2B). Similarly, multivariate binary logistic regression analyses confirmed that a high longest tumor diameter was an independent risk factor for ependymal + (OR = 1.767; 95% CI, 1.453–2.149; *p* < 0.001; Figure 2C). Notably, the longest tumor diameter was not associated with lateral ventricle penetration of GBM, as indicated by univariate and multivariate binary logistics analyses (*p* < 0.05; Figures 2D,E).

### Observation of the Growth Pattern of GBM *in vivo*

**FIGURE 3:**
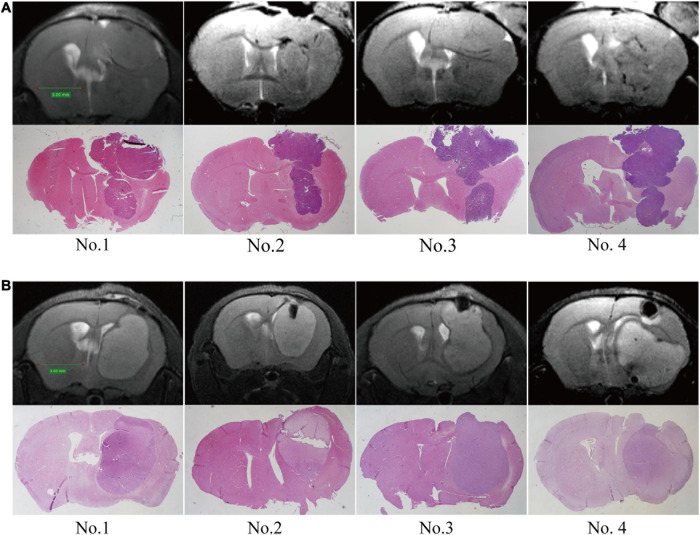
Establishment of orthotopic GBM tumor models. MRI scans were performed at 2 weeks post-implantation, and then upon sacrifice, the brain tissue was further examined by HE staining. **(A)** C57/BL6 mice were inoculated intracranially with GL261 cells. **(B)** Nude mice were inoculated intracranially with U87 cells.

To evaluate the growth pattern of GBM *in vivo*, we next established intracranial tumors in C57/BL6 mice with GL-261 and nude mice with U87. The MRI of orthotopic xenograft tumors was similar to that of patients. The tumor spread along the lining of the lateral ventricle, and the ipsilateral lateral ventricle was compressed and thinned. Following MRI detection, the structure and integrity of the lateral ventricular wall were visualized by HE staining and further supported the MRI results (Figures 3A,B).

### Cell Migration Assay

**FIGURE 4:**
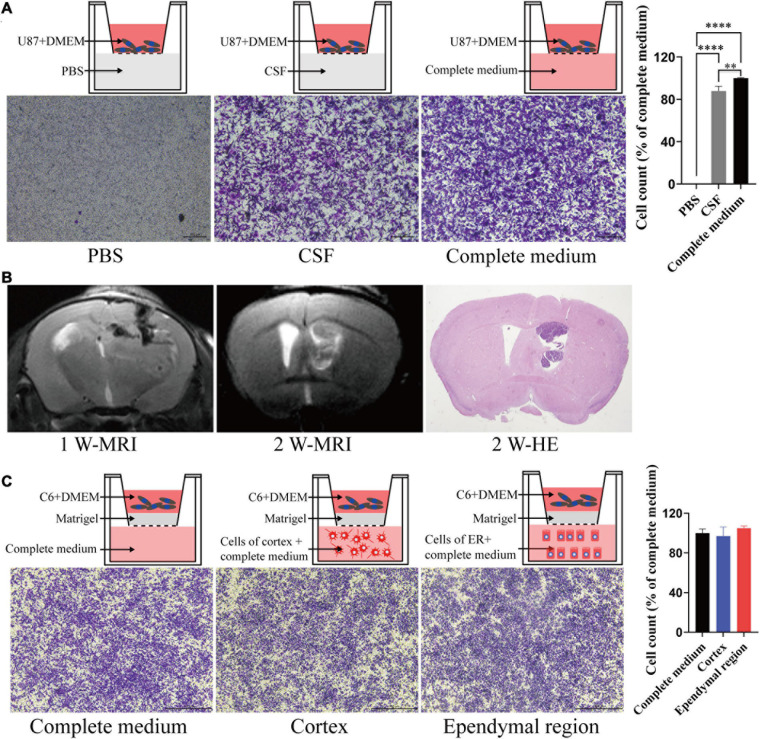
The effects of different components of the cerebral cortex on tumor cell migration and invasion. **(A)** CSF can induce tumor cell migration. **(B)** GL261 cells were injected into the lateral ventricle of C57/BL6 mice; MRI was performed in the first week and second week after injection and then upon sacrifice, and the brain tissue was further examined by HE staining. **(C)** There was no significant difference in the induction of tumor cell invasion between the ependymal region group, cortex group and complete medium group. Migration and invasion were allowed to proceed for 4 h. ^∗∗^*p* < 0.01, ^****^p < 0.0001; ER, ependymal region.

To test the migration and invasion of GBM toward CSF *in vitro*, we utilized a Transwell assay, which allows chemoattraction testing and mimics invasion or migration through an extracellular matrix. We first tested the effect of CSF on GBM migration. Both CSF and complete medium (DMEM with 10% FBS) induced U87 cell migration compared with PBS (*p* < 0.0001), and CSF was only slightly weaker than complete medium (*p* = 0.039; Figure 4A). Then, we penetrated the lateral ventricle of the mice, and GBM cells grew in the lateral ventricle. These results indicated that GBM cells could survive in the lateral ventricle with a low blood supply (Figure 4B). To evaluate the effect of the ependymal region on GBM cell invasion ability, we first placed cells isolated from the ependymal region and cortex of the rats in lower chambers. However, there was no statistically significant difference between the cortex or ependymal region group and the complete medium group (*p* > 0.05; Figure 4C).

**FIGURE 5:**
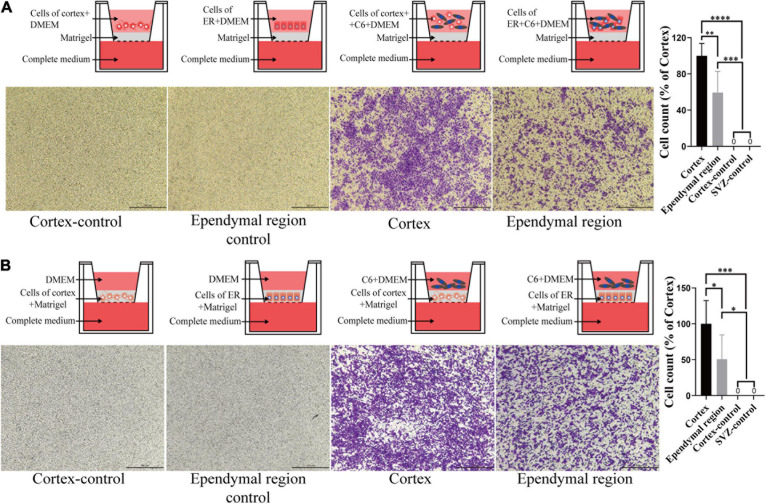
The effect of cell-cell interactions on the invasiveness of GBM cells. **(A)** Compared with that of the cortex group, C6 invasion ability was decreased after coculture with the ependymal region cell suspension, and neither ependymal region nor cortex cells exhibited invasion. **(B)** The invasive ability of C6 in the ependymal region groups was weaker than that in the cortex group when the ependymal region or cortex cell suspension was mixed with Matrigel, and neither ependymal region nor cortex cells exhibited invasion. Invasion was allowed to proceed for 4 h. ^∗^*p* < 0.05, ^∗∗^*p* < 0.01, ^∗∗∗^*p* < 0.001, ^****^p < 0.0001; ER, ependymal region.

To better mimic the microenvironment in which the tumors grow, we set up two coculture systems. We first placed the ependymal region and cortex cell suspension with or without mixing C6 in the upper chamber and added complete medium to the lower chamber. Neither ependymal region nor cortex cells exhibited invasiveness. Interestingly, compared with the C6 cortex cell coculture group, the invasive ability of C6 cells was significantly decreased when they were cocultured with cells from the ependymal region (*p* = 0.0054; Figure 5A). Then, we further mixed the cells from the ependymal region and cortex with Matrigel and solidified them on the bottom of the upper chambers. Consistent with previous results, cells from the ependymal region and cortex were not invasive, and C6 cells in the C6 ependymal region cell coculture group showed reduced invasion compared to the C6 cortex cell coculture group (*p* = 0.0488; Figure 5B).

**FIGURE 6:**
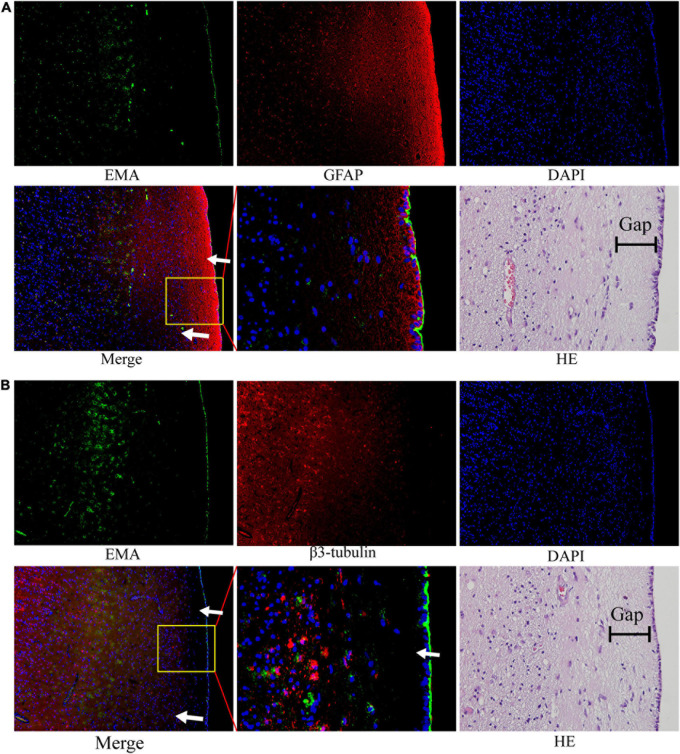
The nature of the subependymal gap was tested by immunofluorescence. All images were taken from the same tissue section. **(A)** EMA (green) was mainly expressed in the ependymal region and to a lesser extent in tumor cells. The gap with high expression of GFAP (red) was visualized around the ependymal region. **(B)** EMA (green) was still expressed in the ependymal region, but very little β3-tubulin expression was observed in the subependymal gap. (The tumor tissue section is from patient No. 4. HE staining corresponds to a similar position as the immunofluorescence image; arrows: subependymal gap).

We noted that ependymal and tumor cells were delimited by a gap around the ependymal region in HE sections. The subependymal gap was strongly positive for GFAP expression but did not express EMA (Figure 6A). EMA was distributed mainly on the ependymal surface and partly in GBM cells (Figure 6A). The pan-neuronal marker β3-tubulin was expressed in tumor cells but was not observed in the ependymal or subependymal gaps (Figure 6B).

## Discussion

The highly invasive nature of GBM is an essential contributes to it extremely poor prognosis and challenging treatment (Tanaka et al., 2013). Generally, the body has a natural ability to combat cancer (Waldman et al., 2020; Wculek et al., 2020). Elucidating the natural antitumor effect may therefore lead to a novel strategy for possible future therapeutic applications.

Ependymal region tissues caught our attention. The ependyma forms important structural barriers between the ventricles and the brain parenchyma. Our clinical data analysis show that it is rare for GBM patients to have tumor cells penetrate into the ventricles while the ependyma remains intact. HE and immunohistochemistry detection verified this phenomenon. Furthermore, we validated our findings *in vivo* in an orthotopic tumor model. GBM propagated along the ependymal in the animal brain, and the growth pattern was consistent with the clinical phenomenon. One possible explanation is that the larger tumor volumes may account for this phenomenon. However, our analysis showed no significant correlation between ventricular entry and tumor volume. Moreover, the ependyma is a thin wall composed of a monolayer of cells. It is therefore unlikely that ependymal region tissues inhibit GBM cell invasion via mechanical forces.

Although CSF fills the brain ventricles, to date, little is known about whether the inhibition of GBM cell invasion is due to the function of CSF. Thus, we assessed the effect of patient-derived CSF on GBM cell migration. Unexpectedly, compared with PBS, both CSF and complete medium could significantly induce U87 cell migration. Therefore, CSF may not be the reason for hindering GBM from entering the ventricle.

A more plausible scenario would be that certain cells located in ependymal region tissues, including ependyma and SVZ, exert a biological effect to suppress tumor cell migration or invasion. However, it is contradictory to what we discussed here because of its key role in tumorigenesis (Altmann et al., 2019; Darazs et al., 2019; Mistry, 2019; Waldman et al., 2020). Most studies have focused on the prognostic impact of tumor-SVZ contact, but the influencing factors of tumor-SVZ contact in patients with GBM are still unclear (Darazs et al., 2019; Mistry et al., 2019). More importantly, few studies have focused on the phenomenon that GBM rarely enters the lateral ventricle, and the role of the ependyma has been ignored (Chen et al., 2015; Spiteri et al., 2019).

According to the current MRI criteria, in most cases (66%), tumors had contact with the ependymal region (Table 1 and Figure 2A). Our results are in line with previous studies by Liu et al., in which most GBM patients’ tumors involved the ependymal region (80%) (Liu et al., 2016). Qin et al. (2017) reported that precursor neuronal cells of the ependymal region may attract tumor cells to migrate to the ventricle, but he did not mention whether the GBM penetrated into the ventricle. Another study suggested that GBM located in proximity to the ependymal region exhibited mRNA expression profiles associated with stem cell properties and increased DNA repair capacity, and that might be associated with GBM-ependymal contact (Lin et al., 2017; Steed et al., 2020). However, Akshitkumar et al. reported that after excluding the volume factor of the sample, there was no significant difference in gene expression between GBM with and without contact with the ependymal region. Thus, for tumors and the SVZ or ependymal region contacted, differential gene expression may be an outcome of volume rather than a precondition.

In this study, logistic regression analysis results showed that tumor diameter was the only independent risk factor for ependymal +, which was consistent with another meta-analysis (Mistry, 2019). This study found that ependymal + GBMs were significantly larger than ependymal- GBMs. Therefore, we suggest that due to the limited space for growth around the GBM, a larger volume was more likely to contact the ependymal region. In addition, their larger volumes and deeper location may also decrease the likelihood of gross total resections, and these findings can partly explain the poor prognosis of ependymal + patients (Pichlmeier et al., 2008; Mcgirt et al., 2009; Chaichana et al., 2014). Therefore, we consider volume to be the key driving factor leading to tumor contact with the ependymal region.

Due to ethical reasons, we cannot obtain human SVZ tissue for *in vitro* experiments, so the establishment of *in vitro* experimental model has become a problem to be considered in this study. Because there may be rejection reactions between tissues and cells of different species (Lu et al., 2019), we think it may be more reasonable to use GBM cells from the same species for transwell experiments. However, the ependymal area in C57/BL6 mice was too small to obtain enough cells while avoiding mixing with cells from the cerebral cortex. Compared with C57/BL6 mice, the ependymal region of Sprague Dawley rat was larger, and the cells were easier to distinguished and collected. Therefore, we finally decided to use rat ependymal cells and C6 (GBM cells of rat origin) for *in vitro* verification experiments.

To eliminate the influence of the physical barrier of the ependymal region, we isolated the ependymal region and cortex from rats and dissociated them into a single-cell suspension (Qin et al., 2017). The cell suspension was mixed with complete medium and placed in the lower chambers to evaluate the effect on GBM cell invasion by a Transwell assay. However, there was no significant difference observed in the ependymal region and cortex groups compared to the complete medium group (Figure 4C). Comparatively, the invasion ability of C6 cells was decreased after coculture with ependymal region cells (Figure 5A). These results suggested that the reduced invasive capacity might be caused by the interaction between ependymal region cells and GBM rather than cell secretion. To further verify this result, the ependymal region or cortex cell suspension was mixed with Matrigel and placed on the bottom of the upper chambers to mimic *in vivo* tumor microenvironments. Consistent with the coculture results, C6 invasion in the ependymal region groups was weaker than that in the cortex groups (Figure 5B), and the results supported the inference that ependymal region cells may inhibit GBM invasion via cell interactions.

We noted that there was a thin gap between the GBM cells and the ependyma by HE staining. The components in this gap seem to block the invasion of tumors into the ependyma and ventricle. Therefore, we assumed that the component of the gap around the ependyma was a key element for the suppression of tumor penetration into the ventricle. This structure around the ependyma has also been described previously (Sanai et al., 2004). The study found that the gap surrounding the ependyma may be composed of the extracellular matrix of astrocytes. Likewise, our results verified that GFAP expression was strongly positive, but interestingly, no β3-tubulin was observed in this structure. Thus, the evidence that this subependymal gap is composed of accessory structures of astrocytes is not yet solid, and further studies are required to determine the specific components and mechanisms of the inhibition of GBM invasion within the ependymal region.

## Conclusion

Overall, ependymal region tissue has an inhibitory effect on the invasion of GBM via a non-mechanical force. However, further studies are required to investigate the cellular and molecular mechanisms by which this inhibition occurs.

## Data Availability Statement

The original contributions presented in the study are included in the article/Supplementary Material, further inquiries can be directed to the corresponding author/s.

## Ethics Statement

The studies involving human participants were reviewed and approved by the Ethics Committee of Nanfang Hospital. The patients/participants provided their written informed consent to participate in this study. The animal study was reviewed and approved by Ethics Committee of Nanfang Hospital.

## Author Contributions

KL, HS, ZL, GY, RY, BN, ZW, TZ, WZ, and XW performed experiments. ZL and GH provided reagents. KL, CW, and YL analyzed data and wrote the manuscript. All authors contributed to the article and approved the submitted version.

## Conflict of Interest

The authors declare that the research was conducted in the absence of any commercial or financial relationships that could be construed as a potential conflict of interest.

## Supplementary Material

Supplementary Figure 1 Representative MRI of the patients with ependymal or ventricle entry. **(A)** MRI image of ependymal-treated patients. The distance between the contrast-enhancing lesion and the lining of the ventricle was more than 2 mm. **(B)** Representative pictures of GBM invasion into the ventricle.
