# Supplementary figures and images for: The Ependymal Region Prevents Glioblastoma From Penetrating Into the Ventricle via a Nonmechanical Force
Source: Front Neuroanat. 2021 Jun 7;15:679405. doi: 10.3389/fnana.2021.679405 (PMC8215287; doi:10.3389/fnana.2021.679405)

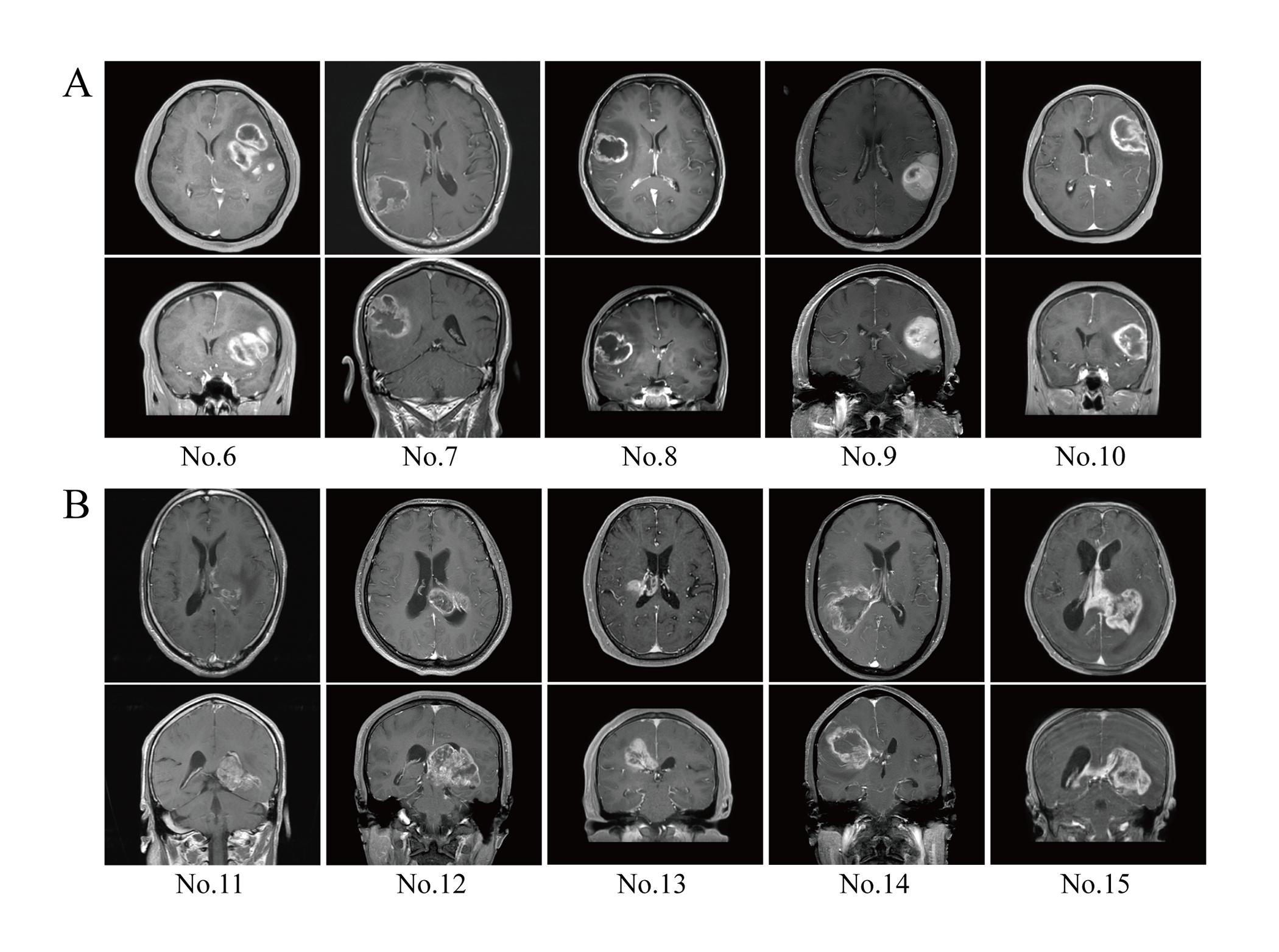

Supplement: Supplementary Figure 1 — Representative MRI of the patients with ependymal or ventricle entry. (A) MRI image of ependymal-treated patients. The distance between the contrast-enhancing lesion and the lining of the ventricle was more than 2 mm. (B) Representative pictures of GBM invasion into the ventricle. [file Image_1.tif]
