# Supplementary material for: The Ependymal Region Prevents Glioblastoma From Penetrating Into the Ventricle via a Nonmechanical Force
Source: Front Neuroanat. 2021 Jun 7;15:679405. doi: 10.3389/fnana.2021.679405 (PMC8215287; doi:10.3389/fnana.2021.679405)
Supplement: Supplementary file 2 [file Table_1.docx]

| Patient no | Gender | Age(years) | Diagnosis | IDH status |
| --- | --- | --- | --- | --- |
| No.1 | Female | 77 | Recurrent GBM | Wild type |
| No.2 | Male | 58 | GBM | Wild type |
| No.3 | Female | 54 | Recurrent GBM | IDH1 mutation |
| No.4 | Female | 51 | Recurrent GBM | Wild type |
| No.5 | Male | 57 | GBM | Wild type |
| No.6 | Male | 52 | GBM | Wild type |
| No.7 | Male | 69 | GBM | Wild type |
| N0.8 | Female | 49 | GBM | Wild type |
| N0.9 | Male | 55 | GBM | Wild type |
| N0.10 | Female | 56 | GBM | Wild type |
| N0.11 | Male | 63 | GBM | Wild type |
| N0.12 | Male | 31 | GBM | Wild type |
| N0.13 | Male | 53 | GBM | IDH1 mutation |
| N0.14 | Male | 57 | GBM | Wild type |
| N0.15 | Male | 77 | GBM | Wild type |
